# Fraudulent Infantile Paralysis “Cures”

**Published:** 1916-09

**Authors:** 


### Fraudulent Infantile Paralysis "Cures."

Officials of the Department of Agriculture charged with the enforcement of the Food and Drugs Act expect that the outbreak of infantile paralysis will tempt unscrupulous persons to offer for sale so-called "cures" or remedies for this dread malady. They, therefore, have issued special instructions to the food and drug inspectors to be particularly alert for interstate shipments or importations of medicines, the makers of which allege that they will cure or alleviate this disease, for which, at the present time, no medicinal cure is known. The officials also warn the public that any preparation put on the market and offered for sale as being effective for the treatment of infantile paralysis should be looked upon with extreme suspicion. Inspectors, accordingly, have been instructed to regard as suspicious, and to collect samples of, all medicines in interstate commerce for which such claims are made. Makers of such fraudulent remedies will be vigorously prosecuted whenever the evidence warrants action under the Sherley amendment to the Food and Drugs Act. So-called remedies for infantile paralysis which are offered for import into the country will be denied entry.

